# Fn14 exacerbates acute lung injury by activating the NLRP3 inflammasome in mice

**DOI:** 10.1186/s10020-022-00514-4

**Published:** 2022-07-30

**Authors:** Xin-Xin Guan, Hui-Hui Yang, Wen-Jing Zhong, Jia-Xi Duan, Chen-Yu Zhang, Hui-Ling Jiang, Yang Xiang, Yong Zhou, Cha-Xiang Guan

**Affiliations:** 1 Department of Physiology, School of Basic Medicine Science, Central South University, Changsha, 410078 Hunan China; 2 Department of Pulmonary and Critical Care Medicine, The Second Xiangya Hospital, Central South University, Changsha, 410011 Hunan China

**Keywords:** Acute lung injury, Fn14, NLRP3 inflammasome, TWEAK, Macrophage

## Abstract

**Background:**

Uncontrolled inflammation is an important factor in the occurrence and development of acute lung injury (ALI). Fibroblast growth factor-inducible 14 (Fn14), a plasma membrane-anchored receptor, takes part in the pathological process of a variety of acute and chronic inflammatory diseases. However, the role of Fn14 in ALI has not yet been elucidated. This study aimed to investigate whether the activation of Fn14 exacerbated lipopolysaccharide (LPS)-induced ALI in mice.

**Methods:**

In vivo, ALI was induced by intratracheal LPS-challenge combined with/without Fn14 receptor blocker aurintricarboxylic acid (ATA) treatment in C57BL/6J mice. Following LPS administration, the survival rate, lung tissue injury, inflammatory cell infiltration, inflammatory factor secretion, oxidative stress, and NLRP3 inflammasome activation were assessed. In vitro, primary murine macrophages were used to evaluate the underlying mechanism by which Fn14 activated the NLRP3 inflammasome. Lentivirus was used to silence Fn14 to observe its effect on the activation of NLRP3 inflammasome in macrophages.

**Results:**

In this study, we found that Fn14 expression was significantly increased in the lungs of LPS-induced ALI mice. The inhibition of Fn14 with ATA downregulated the protein expression of Fn14 in the lungs and improved the survival rate of mice receiving a lethal dose of LPS. ATA also attenuated lung tissue damage by decreasing the infiltration of macrophages and neutrophils, reducing inflammation, and suppressing oxidative stress. Importantly, we found that ATA strongly inhibited the activation of NLRP3 inflammasome in the lungs of ALI mice. Furthermore, in vitro, TWEAK, a natural ligand of Fn14, amplified the activation of NLRP3 inflammasome in the primary murine macrophage. By contrast, inhibition of Fn14 with shRNA decreased the expression of Fn14, NLRP3, Caspase-1 p10, and Caspase-1 p20, and the production of IL-1β and IL-18. Furthermore, the activation of Fn14 promoted the production of reactive oxygen species and inhibited the activation of Nrf2-HO-1 in activated macrophages.

**Conclusions:**

Our study first reports that the activation of Fn14 aggravates ALI by amplifying the activation of NLRP3 inflammasome. Therefore, blocking Fn14 may be a potential way to treat ALI.

## Introduction

Acute lung injury (ALI) and its more severe form, acute respiratory distress syndrome (ARDS), are the most common critical illnesses in clinical practice. ARDS is characterized by the development of uncontrolled pulmonary inflammation, disruptions of the alveolar-capillary barrier function, and non-cardiogenic pulmonary edema (Fan et al. 2018). Particularly, the infiltration of inflammatory cells and increased pro-inflammatory cytokines and chemokines are the major pathological hallmarks of ALI. As the worldwide spread of SARS-CoV2, the research on the mechanism of ARDS is drawing more attention, as severe COVID-19 patients most end in ARDS (Xu et al. 2020). Despite multiple diverse therapeutic methods, only a few effective therapies are available to treat ALI. The mortality in critically ill patients remains at 40% (Matthay et al. 2019). Better understanding the molecular basis of ALI may explore new anti-inflammatory drugs.

The NLRs family, pyrin domain containing 3 (NLRP3) inflammasome, one of the pattern recognition receptors, plays a central role in the development of ALI (Zhang et al. 2016). The NLRP3 inflammasome is a multiprotein complex comprised of NLRP3, pro-caspase-1, and ASC. We have identified that inhibiting the activation of NLRP3 inflammasome alleviates LPS-induced ALI (Yang et al. 2020). The activation of NLRP3 inflammasome includes two steps: priming and activation. Toll-like receptors (TLR) mediate the priming of NLRP3 inflammasome through the nuclear factor-κB (NF-κB) pathway. Damage-related molecular patterns, such as adenosine triphosphate (ATP), mediate the activation of the NLRP3 inflammasome by inducing reactive oxygen species (ROS), ion flux (calcium ion influx or potassium ion outflow), or lysosomal detuning (Swanson et al. 2019). Moreover, the activation of NLRP3 inflammasome promotes the self-cleavage of Caspase-1 precursor into activated Caspase-1 (Caspase-1 p10 and Caspase-1 p20), then mediates the maturation and secretion of IL-1β and IL-18 (Pinar et al. 2020). We have reported that vasoactive intestinal peptides and epoxyeicosatrienoic acids attenuate LPS-induced ALI in mice by targeting the activation of NLRP3 inflammasome (Yang et al. 2020; Zhou et al. 2020; Luo et al. 2020). However, the mechanism underlying the activation of NLRP3 inflammasome in LPS-induced ALI mice needs to be investigated.

Fibroblast growth factor-inducible 14 (Fn14) is the receptor of tumor necrosis factor-like weak inducer of apoptosis (TWEAK) and belongs to the TNF receptor superfamily (Winkles 2008). Fn14 has a low level of ubiquitous expression on many cells. However, stress or inflammation could induce an increased expression. Fn14 is reportedly involved in acute and chronic inflammatory diseases, such as hepatitis, rheumatoid arthritis, and intestinal diseases (Affo et al. 2013; Kuijk et al. 2010; Martino et al. 2016). In addition, the increased expression of Fn14 in the lungs of sepsis patients correlates with tissue damage (Zou et al. 2018). However, whether Fn14 participates in ALI and its mechanism remains unclear. Fn14 activation could induce both the classical (canonical) pathway and the alternative (non-canonical) NF-κB signaling, leading to pro-inflammatory cytokines expression and contributing to tissue inflammation (Sanz et al. 2011). Interestingly, Fn14 promotes the maturation and secretion of IL-1β (Xu et al. 2017). Therefore, we assume that Fn14 participates in the cascade reaction of ALI, which may be related to NLRP3 inflammasome activation.

In this study, we investigated whether blocking Fn14 with an inhibitor (aurintricarboxylic acid, ATA) (Zhang et al. 2019) conferred protection against LPS-induced ALI. The study also could provide new insight into the mechanism underlying the activation of NLRP3 inflammasome in macrophages during ALI.

## Materials and methods

### Animal

Adult (6–8 weeks, 18–20 g) male C57BL/6J mice were purchased from Hunan SJA Laboratory Animal Co., Ltd (Hunan, China). All animal experiments were approved by the Animal Care and Use Committee at Central South University and strictly followed guidelines regarding the care of laboratory animals. All animal experiments were approved by the Ethics Committee of the School of Basic Medical Science, Central South University (2020sydw0685, Changsha, China).

### ALI model and animal treatment

C57BL/6J mice were randomly divided into four groups: (a) the Control group, (b) the ATA group, (c) the ALI group, and (d) the ALI + ATA group. Mice in the Control and ATA groups received 50 µL sterile saline intratracheally. Mice in the ALI and ALI + ATA groups were given an intratracheal injection of LPS (*E. coli* O111:B4; Sigma-Aldrich; USA) at 5 mg/kg dissolved in 50 µL sterile saline. After intratracheal instillation, the mice were kept vertical for 5 min to ensure the distribution of the LPS or sterile in the lungs. In the ATA and ALI + ATA groups, ATA (20 mg/kg) was intraperitoneally injected 1 h prior to the LPS instillation. All mice were sacrificed 12 h after LPS instillation, and the lungs were collected and weighted. For the survival study, the mice were intratracheally injected with LPS at a lethal dose (25 mg/kg). Saline or ATA (20 mg/kg) was intraperitoneally injected into mice 1 h before or after LPS administration. The survival rate was monitored every 6 h. All surgical procedures were performed under 1.0% pentobarbital in saline (80 mg/kg).

### Hematoxylin-eosin (HE) and inflammatory injury analysis

The lung tissue samples were collected, fixed in 10% formalin, embedded in paraffin, sectioned at 4 μm, and stained with HE. Histological scoring was performed blindly in line with four parts: alveolar congestion, hemorrhage, infiltration, and thickness of the alveolar wall/hyaline membrane formation. The lung injury score was measured as previously described (Duan et al. 2017). The severity of the injury was graded from 0 to 4 according to 5 variables: hyaline membranes, neutrophils in the alveolar space, hemorrhage, septal thickening, and pertinacious debris filling the airspaces.

### Lactate dehydrogenase (LDH) assay

The activity of LDH in the serum of mice was determined by a Lactate Dehydrogenase Activity Assay Kit (Jincheng Bioengineering Institute, Nanjing, China). Briefly, the serum samples were incubated with NADH and pyruvate for 15 min at 37 ℃. Then, the enzymatic reaction was stopped by 0.4 mol/L NaOH. Then, absorbance was measured at 450 nm.

### Bronchoalveolar lavage fluid (BALF)

As previously described (Zhou et al. 2017), BALF was collected with 0.8 mL of ice-cold PBS. The BALF was centrifuged at 1500 rpm for 5 min. The pelleted cells were resuspended in PBS, and the cell numbers were counted. Protein concentration in BALF, an indicator of alveolar-capillary permeability, was measured using a BCA kit (Thermo Fisher Scientific). The number of total cells, macrophages, and neutrophils were counted with a hemocytometer and Wright-Giemsa staining.

### Measurement of myeloperoxidase (MPO)

The lung tissue was weighed and homogenized. Then, according to the manufacturer’s instructions, MPO activity in the lungs was measured by the MPO assay kit (Nanjing Jiancheng Bioengineering Institute, China).

### Measurement of superoxide dismutase (SOD) and malondialdehyde (MDA)

About 40 mg of left upper lung tissue was homogenated in cold PBS at a ratio of 1:10 (weight: volume). Protein concentration was determined using the BCA kit. The MDA level was measured by the thiobarbituric acid (TBA) method using a lipid peroxidation MDA assay kit (Jiancheng Biotech, Nanjing, Jiangsu, China) according to the manufacturer’s instructions. Absorbance at 530 nm was measured by the microplate reader. SOD activity was evaluated by the xanthine oxidase method using a SOD activity assay kit (Jiancheng Biotech, Nanjing, Jiangsu, China) according to the manufacturer’s instructions. Absorbance was measured at 450 nm by the microplate reader.

### Measurements of cytokine levels in the lungs

The contents of tumor necrosis factor-alpha (TNF-α), interleukin-1beta (IL-1β), and monocyte chemoattractant protein-1 (MCP-1) in lung tissue, BALF, and cell culture supernatant were determined by specific enzyme-linked immunosorbent assay (ELISA) kits (Invitrogen; Thermo Fisher Scientific) according to the manufacturer’s instructions. The contents of interleukin-18 (IL-18) were determined by specific enzyme-linked immunosorbent assay (ELISA) kits (Jianglai Biotech, Shanghai, China) according to the manufacturer’s instructions.

### Real-time polymerase chain reaction (PCR)

Total RNA was collected from lung tissue using RNAiso Plus (TaKaRa Clontech, Kusatsu, Japan) based on the manufacturer’s instructions. RNA was quantified by measuring absorption at 260 nm. The generation of cDNA from the total RNA (1 µg) was performed with Prime Script RT reagent Kit with gDNA eraser (TaKaRa). Real-time PCR was performed with SYBR Fast qPCR mix (TaKaRa) on a Bio-Rad real-time PCR system (CFX96 Touch™, Bio-Rad, USA). β-actin was used as the internal control. The relative expressions (relative values) of the objective genes were calculated using the 2^−ΔΔCt^ method (Zhang et al. 2020). The sequence of primers used in this study is shown in Table 1.

**Table 1:** Sequences of specific primers used in this study

Gene	Forward primer (5′ to 3′)	Reverse primer (5′ to 3′)
*β-actin*	CACCATGTACCCAGGCATTG	CCTGCTTGCTGATCCACATC
*Mcp-1*	GTCCCTGTCATGCTTCTGG	GCGTTAACTGCATCTGGCT
*Tnf-α*	CCACCCGCTCTTCTGTCTA	TGGTTTGTGAGTGAGGGT
*Pro-Il-1β*	CAGGCAGGCAGTATCACTCA	AGCTCATATGGGTCCGACAG
*Trem-1*	CTGTGCGTGTTCTTTGTC	CTTCCCGTCTGGTAGTCT
*Asc*	GACAGTACCAGGCAGTTCGT	AGTCCTTGCAGGTCAGGTTC
*Nlrp3*	TACGGCCGTCTACGTCTTCT	CGCAGATCACACTCCTCAAA
*Fn14*	CAGATCCTCGTGTTGGGATT	AACTAGAAACCAGCGCCAAA

### Western blot analysis

Total protein was extracted from mouse lungs and cells by RIPA. Protein concentration was detected by the bicinchoninic acid assay (BCA, Thermo Fisher Scientific). Equal amounts of protein (30 µg) were loaded on SDS-PAGE and transferred to a polyvinylidene fluoride membrane (Zhou et al. 2016). After blocking with skim milk (5%) in Tris-buffered saline (TBST) for 1 h at room temperature, the membrane was probed with the primary antibodies at 4 °C overnight: rabbit anti-NLRP3 antibody (1:2000; CST, Danvers, MA), rabbit anti-Nrf2 antibody (1:1000, CST), rabbit anti-HO-1 antibody (1:1000, Abcam), goat anti‐IL‐1β antibody (1:2000; R&D, Minneapolis, MN), rabbit anti‐caspase‐1 antibody (1:1000; Abcam, Eugene, OR), rabbit anti‐ASC antibody (1:1000, CST), rabbit anti-Fn14 antibody (1:1000, Abcam), rabbit anti-β-actin antibody (1:7500, Signal way Antibody, College Park, MD, USA), and rabbit anti‐α‐tubulin antibody (1:7500, Servicebio, China). After incubation with peroxidase‐conjugated secondary antibodies (1:7500, Signal way Antibody) at room temperature for 1 h, the signal was developed using an ECL chemiluminescence kit (Millipore, USA). The band intensities were quantitated using Image Lab software and normalized to internal reference values accordingly.

### Isolation and treatment of primary murine peritoneal macrophages

The isolation and culture of primary murine peritoneal macrophages were conducted as described (Zhong et al. 2019). Four days after the intraperitoneal injection of 3 mL 3% thioglycolate (Sigma-Aldrich) into C57BL/6J mice, peritoneal macrophages were harvested by peritoneal lavage with precooled Roswell Park Memorial Institute RPMI 1640 (Gibco, Life Technologies, Carlsbad, CA). The cells were collected by centrifuging at 1000 rpm for 8 min and washed with cooled phosphate‐buffered saline. The cells were resuspended in cell culture medium and counted on the counting plate after sufficient mixing. Macrophages were plated into 6-well or 12‐well plates (2 × 10^6^ cells/well or 1 × 10^6^ cells/well). To investigate the effect of rTWEAK on the macrophages, the cells were treated with rTWEAK (100 ng/mL) and LPS (100 ng/mL) for 12 h. To investigate the activation of the NLRP3 inflammasome, the cells were primed with rTWEAK (100 ng/mL) and LPS (100 ng/mL) for 135 min. Cells were then stimulated with ATP (2.5 mM, Solarbio, China) for 45 min.

### Measurement of ROS

The primary peritoneal macrophages (2 × 10^6^ cells/well) were seeded into the 6-well plates. Dilute DCFH-DA (Nanjing Jiancheng Bioengineering Institute, China) with RPMI 1640 at the ratio of 1:1000 to a final concentration of 10 µM. Add 1 mL diluted DCFH-DA solution to each well and incubate for 20 min at 37 ℃. The cell suspension was obtained by adding 1 mL RPMI 1640, then centrifuged at 1500 rpm for 5 min, followed by resuspension with 400 µL PBS for flow cytometry assay (BD, USA).

### Silenced the expression of Fn14 by shRNA

The short hairpin RNA (shRNA) of Fn14 was purchased from Genechem (Shanghai, China). Fn14 shRNA sequences were gattcggcttggtgttgatgc. Macrophages were infected with concentrated lentivirus. The supernatant was replaced with a complete culture medium after 16 h. After being treated with shRNA, the cells were primed with LPS (100 ng/mL) for 135 min. Cells were then stimulated with ATP (2.5 mM, Solarbio, China) for 45 min.

### Statistical analysis

All experiments were independently repeated three times. All Data were presented as means ± SEM. All data analyses were conducted using SPSS 21. Pearson correlations were estimated to examine linear associations between the protein expression of Fn14 and the inflammation score, the protein expression of TNF-α, and the protein expression of IL-1β. Differences between the two groups were analyzed with a *t*-test. Differences between multiple groups were analyzed using ANOVA, followed by Tukey’s post hoc test. The survival rate was assayed by the log-rank test. A value of *P* < 0.05 was considered significant.

## Results

### Fn14 expression is increased in the lungs of ALI mice

**Fig. 1:**
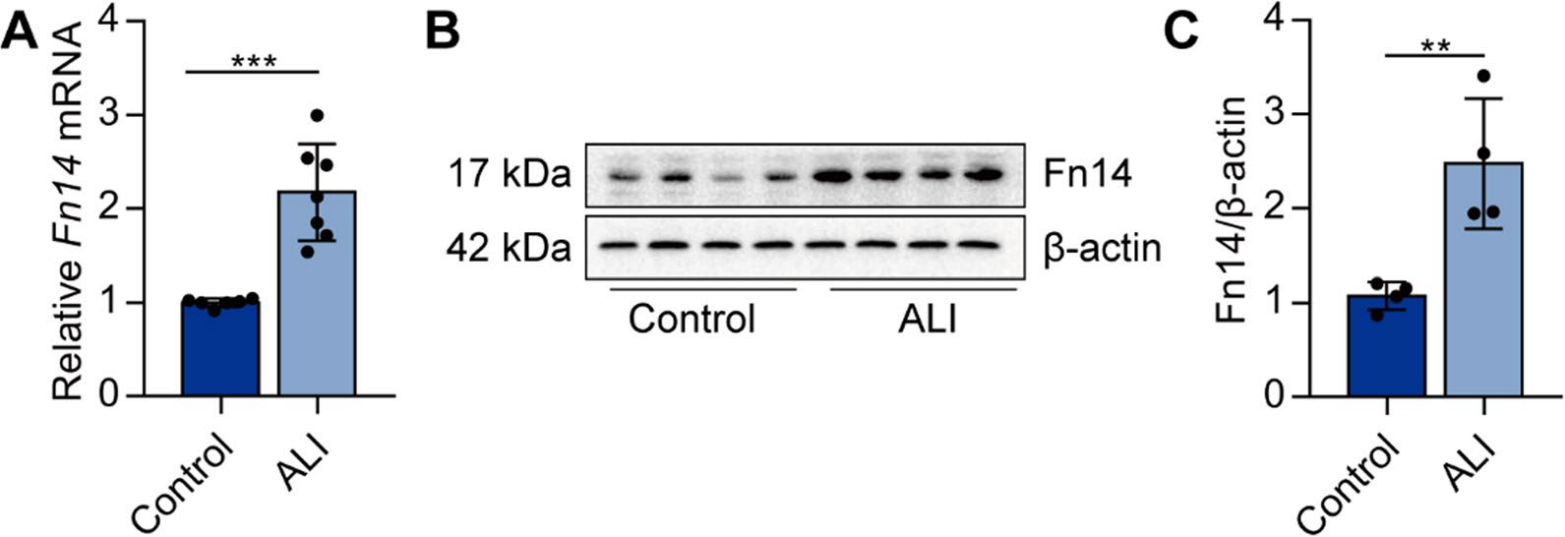
Fn14 expression is increased in the lungs of ALI mice. C57BL/6J mice were intratracheally injected with LPS (5 mg/kg, *i.t.*). Twelve hours later, the expression of *Fn14* mRNA in the lungs was determined by real-time PCR (**A,** *n* = 7). The protein expression of Fn14 in the lungs was determined by Western blot (**B**, **C**, *n* = 4). Data are expressed as the mean ± SD. Differences between the two groups were determined by an unpaired *t*-test. **P < 0.01 and ***P < 0.001

We first investigated whether Fn14 expression was correlated with ALI. The mRNA and protein expression of Fn14 was raised in the lungs of LPS-induced ALI mice (Fig. 1A–C). These data suggest that Fn14 may be a key orchestrator in the pathogenesis of ALI.

### Blockade of Fn14 by ATA alleviates LPS-induced lung injury in mice

**Fig. 2:**
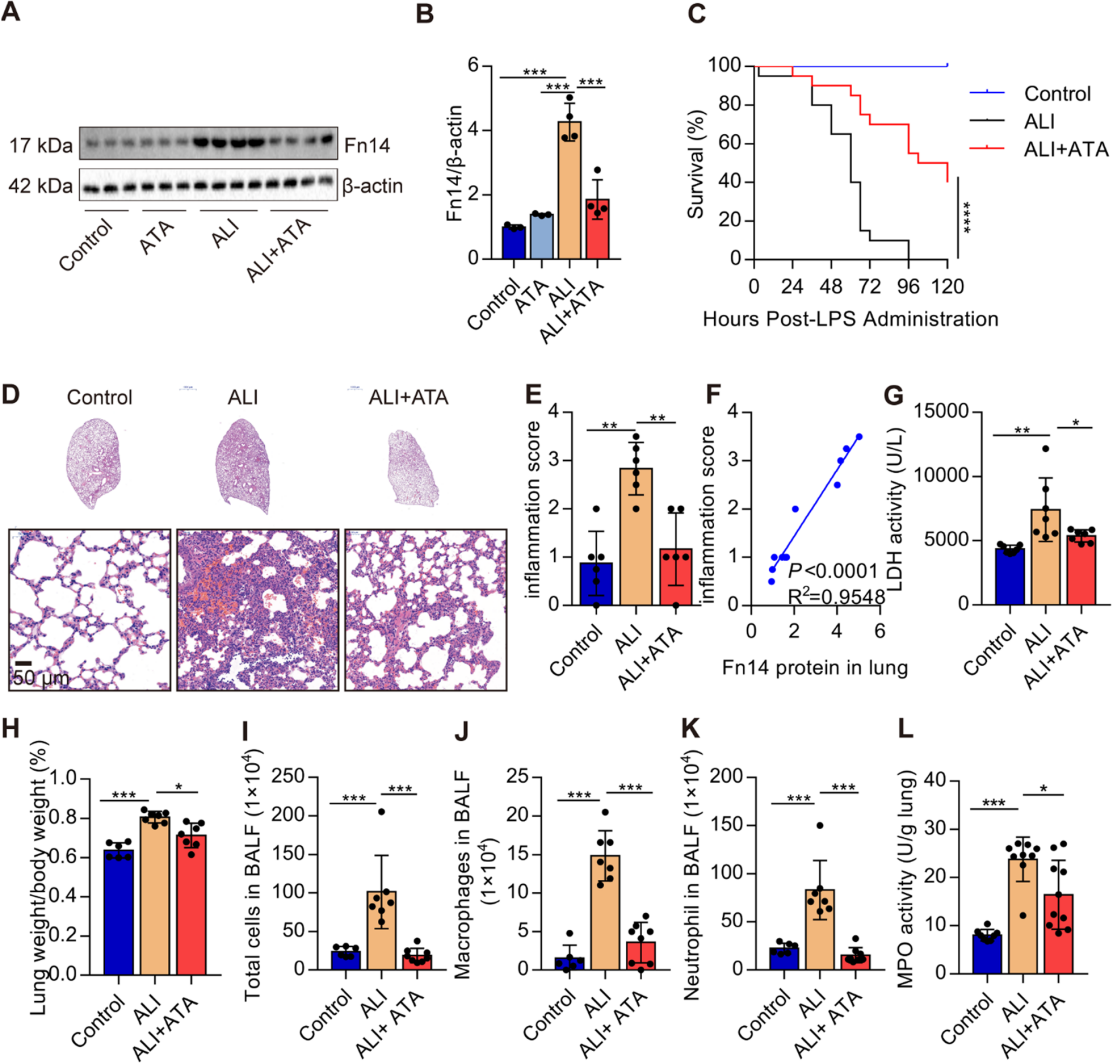
Blockade of Fn14 by ATA alleviates LPS-induced ALI in mice. ATA (20 mg/kg) was administered to mice 1 h before induction of ALI by LPS (5 mg/kg). **A**, **B** The protein expression of Fn14 in the lungs was determined by Western blot (*n* = 4). **C** The mortality was monitored every 6 h over 120 h (*n* = 20 per group). C57BL/6J mice were treated with saline or ATA (20 mg/kg). One hour later, mice were given saline or LPS (5 mg/kg, *i.t.*). **D** Twelve hours later, the lung histopathological change was determined by H&E staining (Bar = 50 μm). **E** The change in lung inflammation score was determined (*n* = 6). **F** The linear associations between the expression of Fn14 protein and inflammation score were analyzed with Pearson correlations analysis. **G** The activity of LDH in BALF was determined (*n* = 6–7). **H** The lung weight/body weight ratio was determined (*n* = 6–7). **I–K** The Quantification of total cells, macrophages, and neutrophils in BALF was determined (n = 6–7). **L** The activity of MPO in lung tissue was determined (*n* = 6–10). Data are expressed as the mean ± SD. Differences among multiple groups were performed using ANOVA. Tukey’s test was used as a post hoc test to make pair-wise comparisons. Survival data were analyzed using the log-rank test. *P < 0.05, **P < 0.01, and ***P < 0.001

ATA was previously identified as a novel inhibitor of Fn14 signaling (Zhang et al. 2019). Here, we adopted ATA to investigate the Fn14 function in ALI. The protein expression of Fn14 in the lungs of ALI mice was downregulated after pre-treatment with ATA (20 mg/kg) 1 h before LPS administration (Fig. 2A, B). Administration of ATA significantly reduced the mortality of mice receiving 25 mg/kg LPS (Fig. 2C). H&E staining indicated the accumulation of inflammatory cells, destruction of the alveolar histological structure, and the collapse of the alveoli in LPS-treated mice. Remarkably, ATA pre-treatment alleviated those pathological changes (Fig. 2D, E). The inflammation score was positively correlated with the Fn14 protein (Fig. 2F). LDH release is positively related to cellular damage. We found that ATA pre-treatment pronounced reduced the LDH activity in the serum (Fig. 2G). In parallel, the W/D ratio of lung tissue was significantly decreased in ATA-treated mice (Fig. 2H). ATA pre-treatment robustly reduced the number of total cells, macrophages, and neutrophils in the BALF (Fig. 2I–K) and MPO activity in the lungs (Fig. 2L), which reflects neutrophil accumulation in the lungs. Taken together, these data indicate that blockade of Fn14 mitigates lung damage in ALI mice.

### Blockade of Fn14 by ATA inhibits the inflammatory responses and oxidative stress in ALI mice

**Fig. 3:**
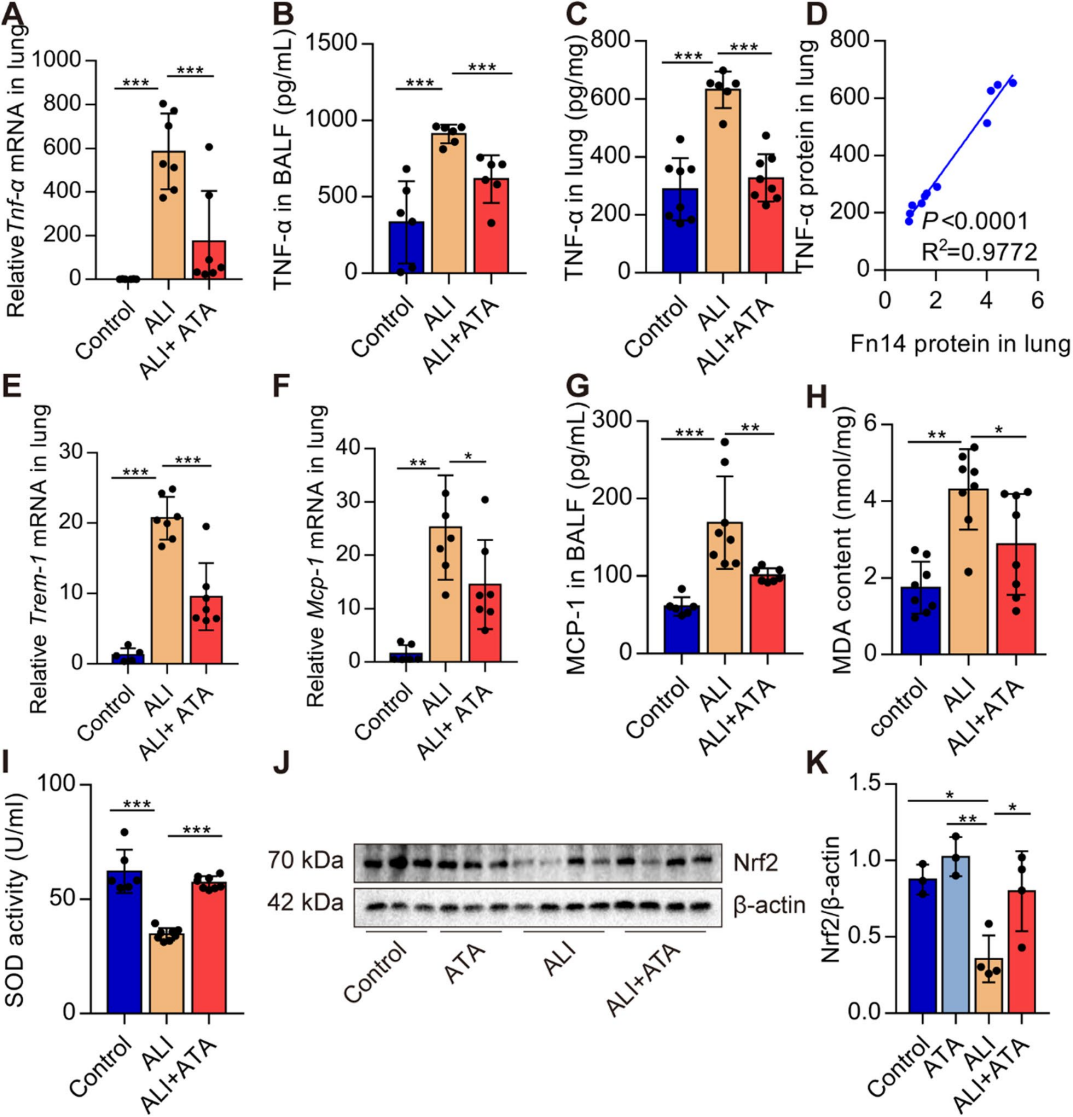
Blockade of Fn14 by ATA reduces the inflammatory responses and oxidative stress in ALI mice. C57BL/6J mice were treated with saline or ATA (20 mg/kg). One hour later, mice were given saline or LPS (5 mg/kg, *i.t.*). **A–C** Twelve hours later, TNF-α mRNA and protein levels were determined by real-time PCR and ELISA (*n* = 6–9). **D** The linear associations between the expression of Fn14 protein and TNF-α protein were analyzed with Pearson correlations analysis. **E** The expression of *Trem-1* mRNA was determined by real-time PCR (*n* = 5–7). **F**, **G** The MCP-1 mRNA and protein were determined by real-time PCR and ELISA (*n* = 6–8). **H** The content of MDA in the lungs was determined (*n* = 8).**I** The activity of SOD in lung tissue was determined (*n* = 6–8). **J**, **K** The protein expression of Nrf2 in the lungs was determined by Western blot (*n* = 3–4). Data are expressed as the mean ± SD. Differences among multiple groups were performed using ANOVA. Tukey’s test was used as a post hoc test to make pair-wise comparisons. *P < 0.05, **P < 0.01, and ***P < 0.001

Inflammation and oxidative stress are dispensable characteristics of ALI. We found that ATA pre-treatment reduced the pro-inflammatory cytokines and chemokines mRNA expression, including *Tnf-α* and *Trem-1* (Fig. 3A, E). Protein expression of TNF-α in BALF and lungs was decreased in ATA-treated mice (Fig. 3B, C). TNF-α protein was positively correlated with Fn14 protein (Fig. 2D). MCP-1 recruits monocytes to the sites of inflammation. MCP-1 mRNA and protein expression were also down-regulated by ATA treatment (Fig. 3F, G). In addition, ATA pre-treatment reduced the MDA formation in the lungs of ALI mice (Fig. 3H). In contrast, ATA pre-treatment remarkably restored the activity of the antioxidative enzyme SOD in the lungs (Fig. 3I). Nuclear factor-erythroid 2-related factor 2 (Nrf2) is a key transcription factor in the antioxidative stress response of cells. ATA pre-treatment remarkably increased the Nrf2 protein expression (Fig. 3J, K). Collectively, these results suggest that blockade of Fn14 inhibits inflammatory responses and oxidative stress in LPS-induced ALI mice.

### Blockade of Fn14 by ATA improves the survival of LPS-treated mice

**Fig. 4:**
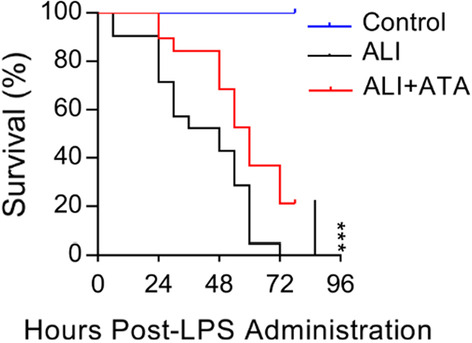
Therapeutic blockade of Fn14 by ATA improves the survival of LPS-treated mice. ATA (20 mg/kg) was administered to mice 2 h after induction of ALI by LPS (25 mg/kg). The mortality of the mice was monitored every 6 h over 96 h (*n* = 20). Survival data were analyzed using the log-rank test. ***P < 0.001

To explore the therapeutic potential of ATA, we investigated the potential benefits of ATA treatment (20 mg/kg) 2 h post-LPS administration. We found that treatment with ATA 2 h post-LPS exposure also significantly improved the survival rate of LPS-treated mice (Fig. 4). This result indicates that blockade of Fn14 shows therapeutic benefits to LPS-induced ALI in mice.

### Blockade of Fn14 by ATA inhibits the NLRP3 inflammasome activation in ALI mice

**Fig. 5:**
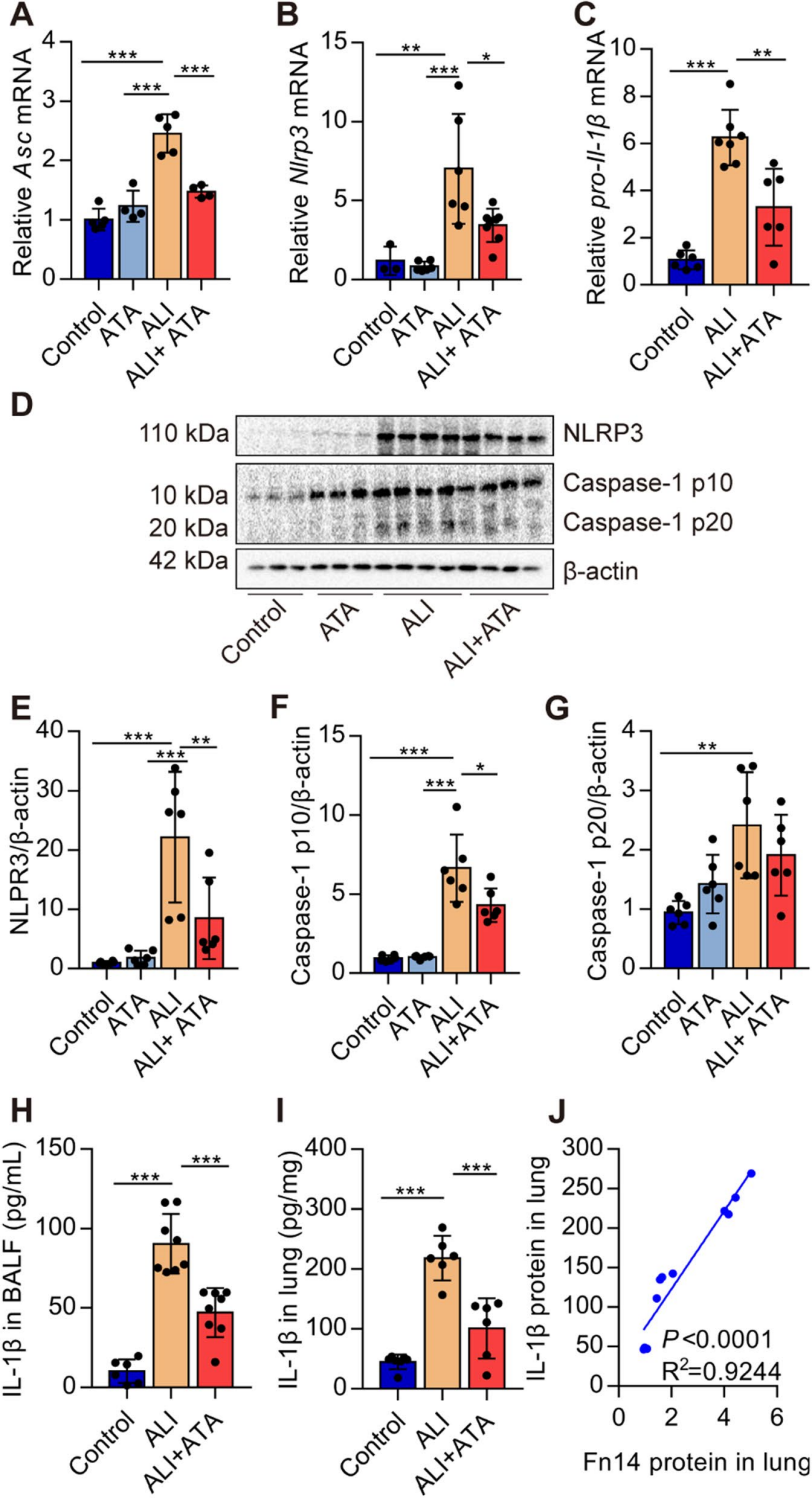
Blockade of Fn14 by ATA inhibits the NLRP3 inflammasome activation in ALI mice. Adult C57BL/6J mice were treated with saline or ATA (20 mg/kg). One hour later, mice were given saline or LPS (5 mg/kg, *i.t.*). **A**–**C** Twelve hours later, the expression of *Asc*, *Nlrp3*, and *pro-Il-1β* mRNA in the lungs was determined by real-time PCR (*n* = 3–7). **D**–**G** The levels of NLRP3, Caspase-1 p10, and Caspase-1 p20 protein in the lungs were determined by Western blot (*n* = 6). **H**–**I** The content of IL-1β protein in BALF and lung tissue was determined by ELISA (*n* = 6–8). **J** The linear associations between the expression of Fn14 protein and IL-1β protein were analyzed with Pearson correlations analysis. Data are expressed as the mean ± SD. Differences among multiple groups were performed using ANOVA. Tukey’s test was used as a post hoc test to make pair-wise comparisons. *P < 0.05, **P < 0.01, and ***P < 0.001

The NLRP3 inflammasome plays a vital role in the pathogenesis of ALI (Yang et al. 2020). We first investigated whether blockade of Fn14 with ATA repressed the activation of NLRP3 inflammasome in vivo. Results showed that ATA profoundly reduced the expression of *Asc*, *Nlrp3*, and *pro-Il-1β* mRNA in the lungs of ALI mice (Fig. 5A–C). Upon NLRP3 inflammasome activation, pro-caspase-1 is hydrolyzed into Caspase-1 p10 and Caspase-1p20, cleaving pro-IL-1β into IL-1β. We found that ATA remarkably decreased the NLRP3, Caspase-1 p20, and Caspase-1 p10 protein levels in the lungs of ALI mice (Fig. 5D–G). NLRP3 inflammasome activation gives rise to the secretion of active IL-1β. The results showed that ATA pre-treatment effectively reduced the levels of IL-1β in BALF and lungs of ALI mice (Fig. 5H, I). IL-1β protein was positively correlated with Fn14 protein (Fig. 2J). These data suggest that the ATA inhibits the activation of NLRP3 inflammasome in the lungs of ALI mice.

### Activation of Fn14 by rTWEAK promotes the priming of NLRP3 inflammasome in macrophages

**Fig. 6:**
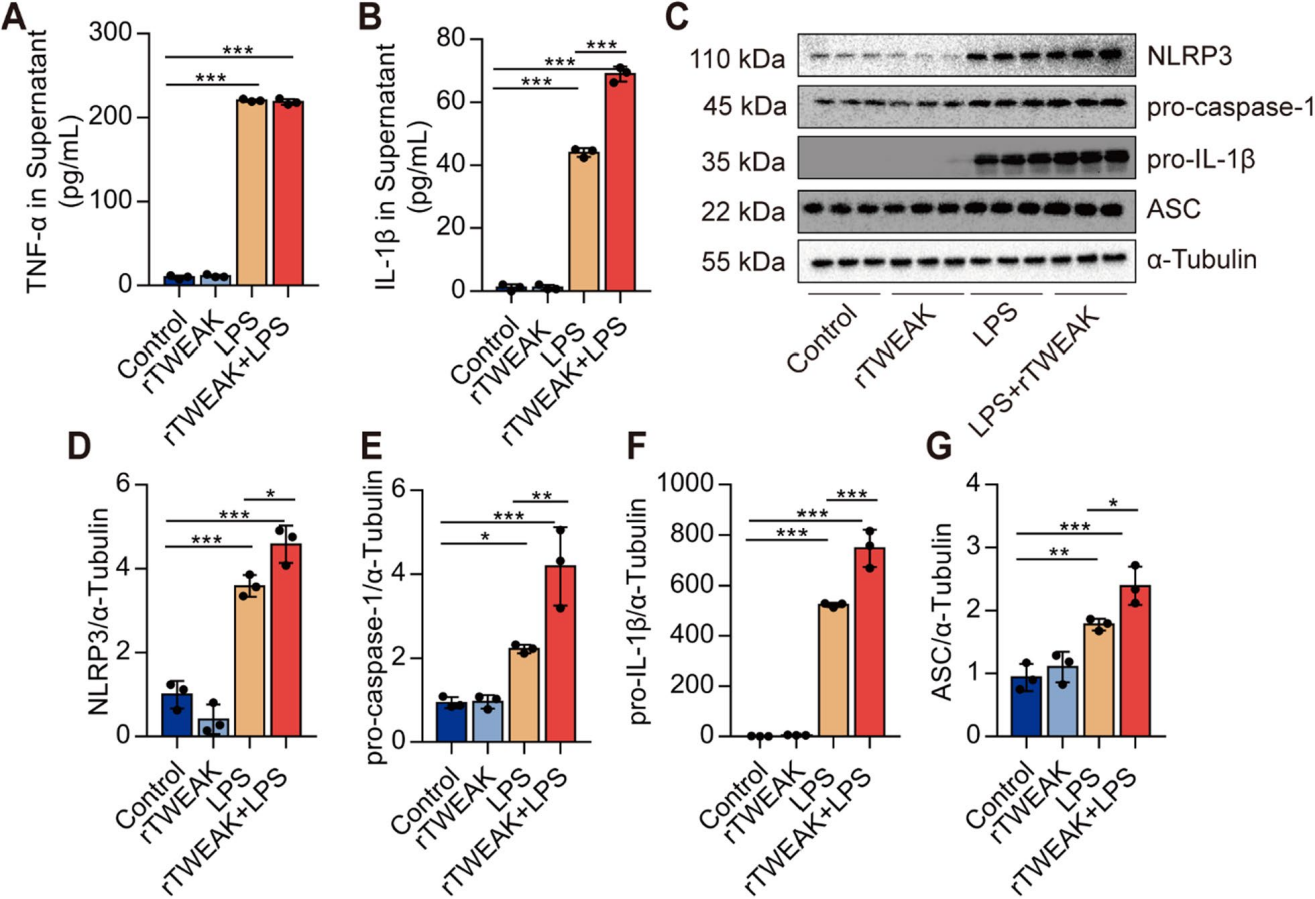
Activation of Fn14 by rTWEAK promotes the priming of NLRP3 inflammasome in macrophages. rTWEAK (100 ng/mL) was added to LPS (100 ng/mL)-challenged primary macrophages for 12 h. **A**, **B** The contents of TNF-α and IL-1β in the supernatant of primary macrophages were determined (*n* = 3). **C**–**G** The expressions of NLRP3, pro-caspase-1, pro-IL-1β, and ASC protein in primary macrophages were determined by Western blot (*n* = 3). Data are expressed as the mean ± SD. Differences among multiple groups were performed using ANOVA. Tukey’s test was used as a post hoc test to make pair-wise comparisons. *P < 0.05, **P < 0.01, and ***P < 0.001

Next, we investigated the effects of Fn14 on the NLRP3 inflammasome priming in primary macrophages. rTWEAK, a natural ligand of FN14, was used to stimulate primary peritoneal macrophages. Interestingly, we found that, compared with the LPS group, rTWEAK significantly increased the secretion of IL-1β but not TNF-α (Fig. 6A, B), indicating that rTWEAK specifically amplifies NLRP3 inflammasome-mediated inflammatory response. Further, we found that LPS induced the upregulations of NLRP3, pro-caspase-1, pro-IL-1β, and ASC expression. The rTWEAK treatment significantly increased the expressions of NLRP3, pro-caspase-1, pro-IL-1β, and ASC (Fig. 6C–G). These results suggest that activation of Fn14 promotes the priming of NLRP3 inflammasome.

### rTWEAK pre-treatment aggravates the activation of NLRP3 inflammasome in macrophages

**Fig. 7:**
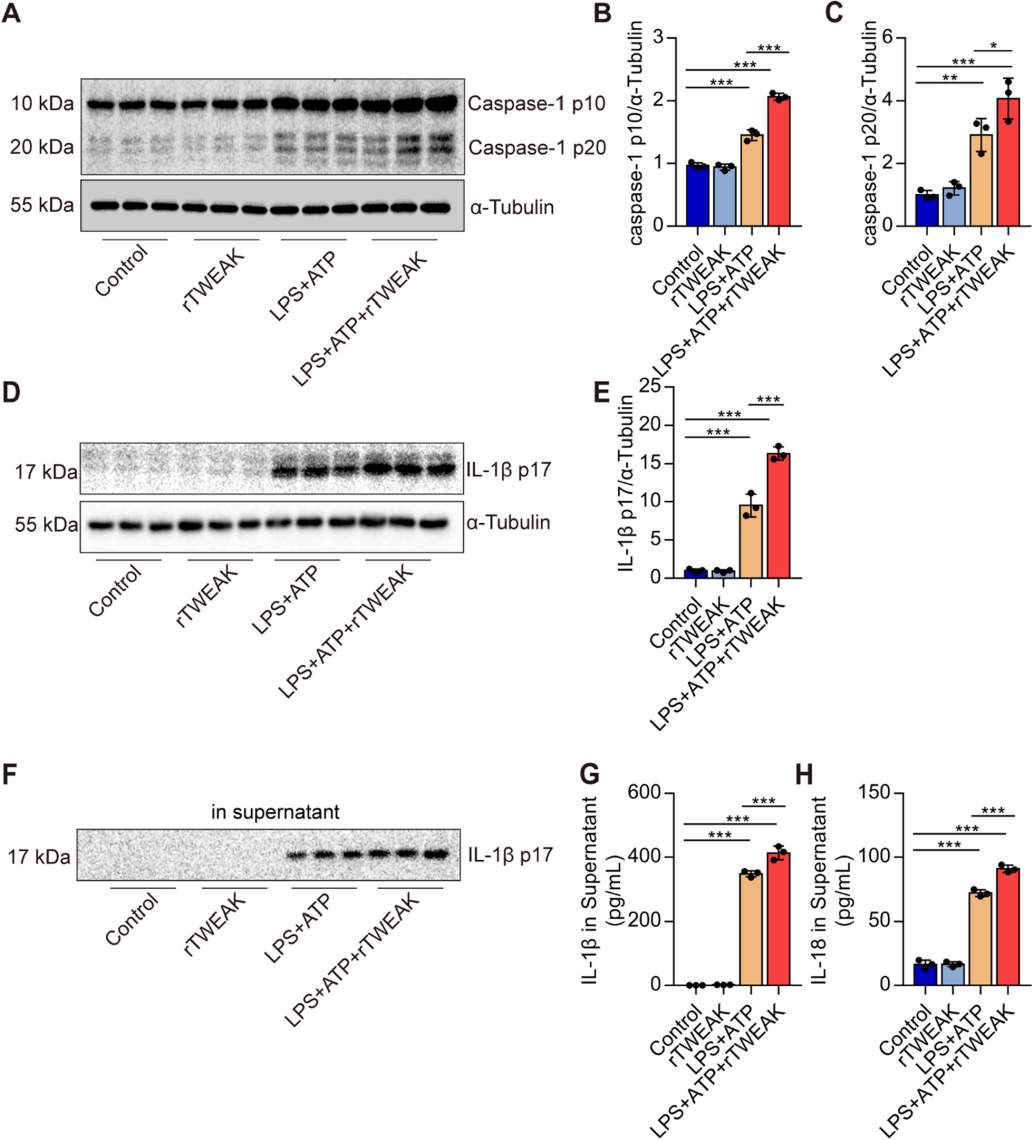
Pre-treated with rTWEAK aggravates the activation of NLRP3 inflammasome in macrophages. The cells were stimulated by rTWEAK (100 ng/mL) and LPS (100 ng/mL) for 135 min, and then ATP (2.5 mM) challenged primary macrophages for 45 min. **A**–**C** The levels of Caspase-1 p10 and Caspase-1 p20 in primary macrophages were determined by Western blot (*n* = 3). **D**, **E** The level of IL-1β p17 in primary macrophages was determined by Western blot (*n* = 3). **F** The level of IL-1β p17 in the supernatant of primary macrophages was determined by Western blot (*n* = 3). **G** The content of IL-1β p17 protein in the supernatant of primary macrophages was determined by ELISA (*n* = 3). **H** The content of IL-18 protein in the supernatant of primary macrophages was determined by ELISA (*n* = 3). Data are expressed as the mean ± SD. Differences among multiple groups were performed using ANOVA. Tukey’s test was used as a post hoc test to make pair-wise comparisons. *P < 0.05, **P < 0.01, and ***P < 0.001

**Fig. 8:**
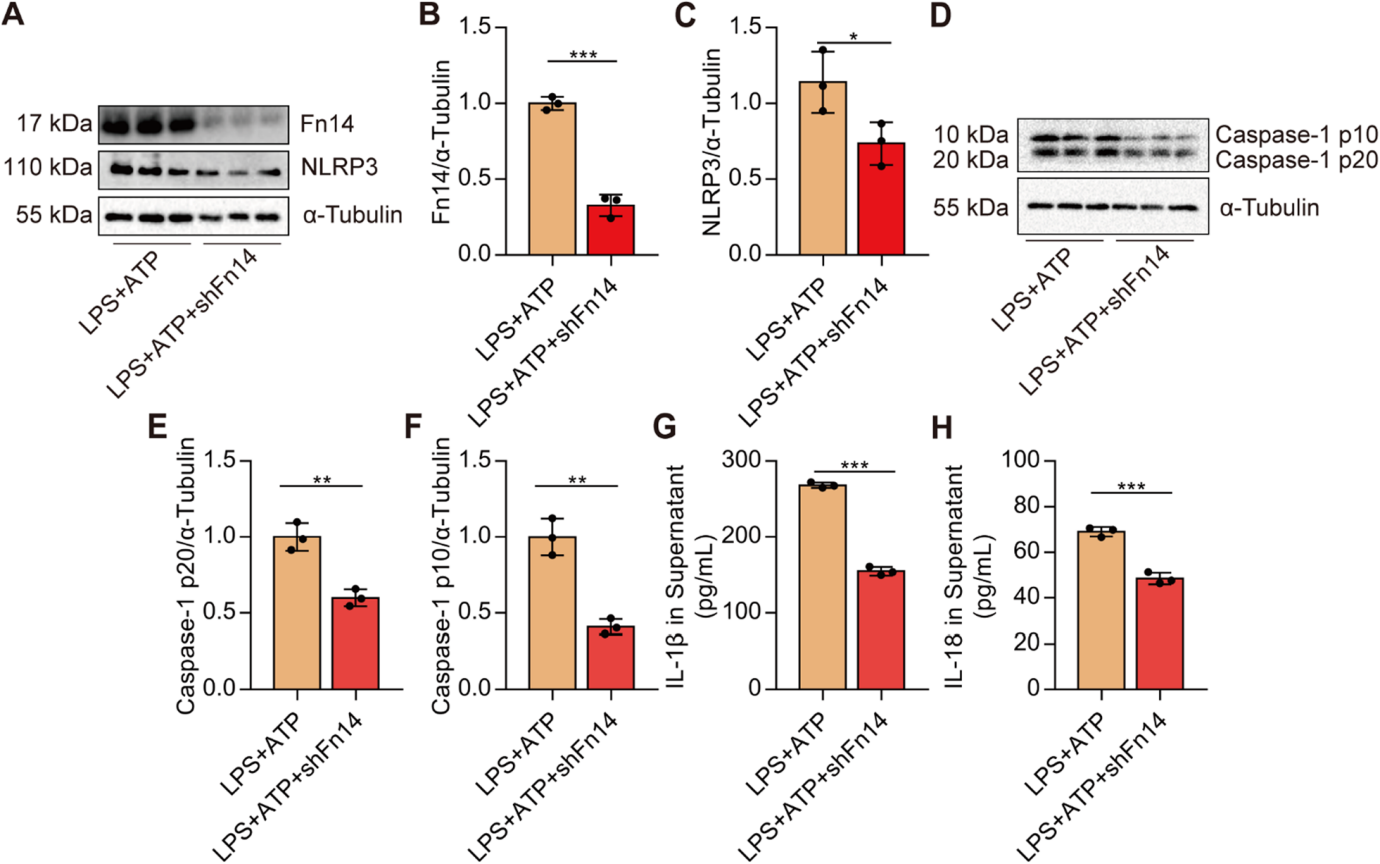
The silence of Fn14 blocks the activation of NLRP3 inflammasome in macrophages. Fn14 shRNA was added before LPS (100 ng/mL)-challenged primary macrophages for 96 h. **A–C** The expressions of Fn14 and NLRP3 protein in primary macrophages were determined by Western blot (*n* = 3). **D–F** The levels of Caspase-1 p20 and p10 in primary macrophages were determined by Western blot (*n* = 3). **G** The contents of IL-1β in the supernatant of primary macrophages were determined (*n* = 3). **H** The contents of IL-18 in the supernatant of primary macrophages were determined (*n* = 3). Data are expressed as the mean ± SD. Differences between the two groups were determined by an unpaired *t*-test. *P < 0.05, **P < 0.01, and ***P < 0.001

We treated macrophages with LPS plus ATP to activate the NLRP3 inflammasome. We found that pre-treatment of rTWEAK significantly increased the levels of Caspase-1 p10 and Caspase-1 p20 in macrophages treated by LPS with ATP (Fig. 7A–C). IL-1β p17 levels in the cells and supernatant increased upon rTWEAK treatment (Fig. 7D-G). IL-18 level in the supernatant was also increased upon rTWEAK stimulation (Fig. 7H). In addition, we silenced the expression of Fn14 in macrophages. Fn14 shRNA significantly decreased the Fn14 and NLRP3 expression induced by LPS and ATP (Fig. 8A–C). Furthermore, the level of Caspase-1 p10 and Caspase-1 p20 was effectively blocked by Fn14 shRNA in macrophages (Fig. 8D–F). IL-1β and IL-18 production were effectively blocked by Fn14 shRNA in macrophages (Fig. 8G, H). These data collectively suggest that activation of Fn14 by rTWEAK aggravates the activation of NLRP3 inflammasome in macrophages.

### rTWEAK pre-treatment promotes the ROS production and reduces the activation of Nrf2-HO-1 in macrophages

**Fig. 9:**
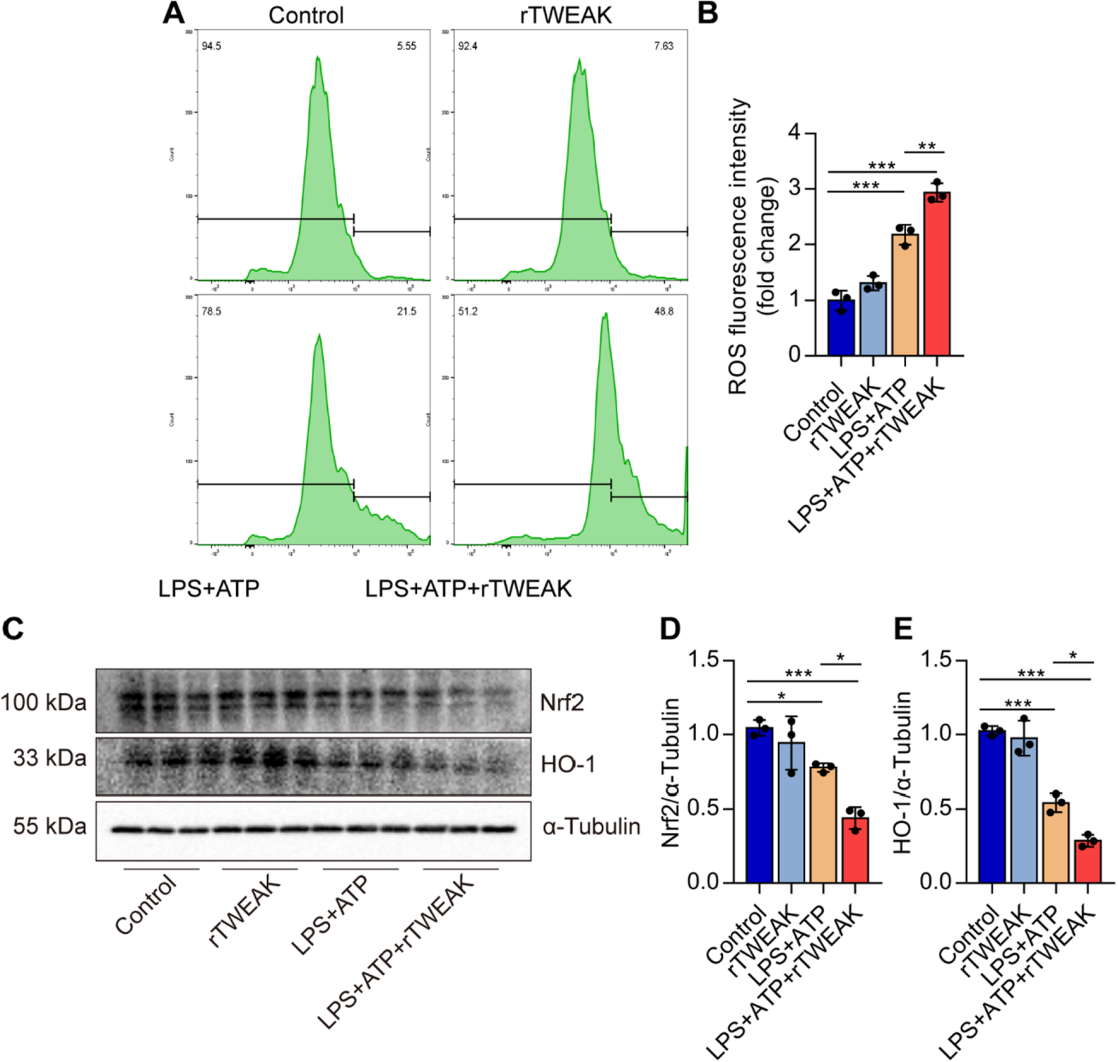
Pre-treated with rTWEAK aggravates the ROS production in macrophages. rTWEAK (100 ng/mL) was added to LPS (100 ng/mL) for 135 min and ATP (2.5 mM)-challenged primary macrophages for 45 min. **A**, **B** ROS production in the primary macrophages was analyzed by the flow cytometry assay (*n* = 3). **C–E** The expressions of Nrf2 and HO-1 protein in primary macrophages were determined by Western blot (*n* = 3). Data are expressed as the mean ± SD. Differences among multiple groups were performed using ANOVA. Tukey’s test was used as a post hoc test to make pair-wise comparisons. *P < 0.05, **P < 0.01, and ***P < 0.001

Given that excessive ROS mediates NLRP3 inflammasome activation, we investigated whether activation of Fn14 could increase ROS production. As demonstrated in Fig. 9, after treatment with LPS + ATP, an increase in the DCF fluorescence intensity was observed in primary macrophages pre-treatment of rTWEAK significantly increased the production of ROS in the primary macrophages (Fig. 9A, B). rTWEAK pre-treatment remarkably decreased the Nrf2 and HO-1 protein expression (Fig. 9C–E). The results confirm that rTWEAK promotes ROS production and inhibits the activation of Nrf2-HO-1, an antioxidant signaling pathway, contributing to the activation of NLRP3 inflammasome in primary macrophages.

## Discussion

In this study, we found blocking Fn14 reduced LPS-induced ALI in mice. Blockade of Fn14 suppressed the activation of NLRP3 inflammasome in vivo. In vitro, Fn14 activation amplified the activation of NLRP3 inflammasome in primary murine macrophages. Our study implies that Fn14 may play a key role in the pathogenesis of ALI and provides a novel mechanism of Fn14 activation on the inflammatory cascade.

This study identifies that elevated Fn14 is a novel mechanism in the uncontrolled inflammatory cascade during ALI. Fn14 is a type I transmembrane protein encoded by fibroblast growth factors-regulating early response gene (Mendez-Barbero et al. 2020). Studies have reported that Fn14 is significantly up-regulated in inflammation-related diseases, such as intestinal diseases and kidney disease, accelerating the course of illness (Dohi et al. 2009; Poveda et al. 2021). Our study found that Fn14 expression was significantly increased in LPS-induced ALI in mice. ATA is an inhibitor of Fn14, which blocks the TWEAK/Fn14 signal axis by inhibiting the binding of TRAF2 (Zhang et al. 2019). Herein, we found that ATA reduced lung tissue pathology injury, and decreased the number of inflammatory cells and pro-inflammatory cytokines such as TNF-α and MCP-1 in BALF. More importantly, the blockade of Fn14 activation effectively improved the survival rate of mice treated with a lethal dose of LPS. These results suggest that blocking Fn14 activation alleviates the LPS-induced ALI. In another study, blockade of Fn14 on pulmonary microvascular endothelial cells (PMVECs) shows a protective effect against cecal ligation and puncture-induced ALI (Zou et al. 2018). We speculated, therefore, that Fn14 may be a novel target for the treatment of ALI.

We reveal a new function of Fn14 in macrophages. Fn14 could express in different types of cells, such as macrophages, fibroblast (Zhang et al. 2021), PMVECs (Zou et al. 2018), vascular smooth muscle cells (Munoz-Garcia et al. 2011), and cancer cells (Dwyer et al. 2021). Previous studies have reported that Fn14 expressed on macrophages promotes oxidative stress through NADPH oxidase activation (Madrigal-Matute et al. 2015) or increases HMGB1 expression and secretion (Moreno et al. 2013). Herein, we found that Fn14 amplifies the activation of NLRP3 inflammasome in macrophages. Blocking Fn14 activation inhibited the expression of NLRP3 inflammasome components, including NLRP3, ASC, pro-caspase-1, pro-IL-1β, and secretion of IL-1β in LPS-induced ALI mice. The accumulation of evidence points to the central role of NLRP3 inflammasome in the ALI (Zhang et al. 2016; Liu et al. 2016). Interestingly, activation of Fn14 with rTWEAK fails to increase the TNF-α secretion, indicating that Fn14 specifically amplifies NLRP3 inflammasome-mediated inflammatory response. Therefore, we propose for the first time that activated Fn14 aggravates pulmonary inflammation by activating NLRP3 inflammasome, which may be a new mechanism explaining the uncontrolled inflammation in ALI.

Oxidative stress is the crucial pathogenesis of ALI. TWEAK-Fn14 axis is directly involved in oxidative stress. Our results indicate that ATA alleviates oxidative stress in the lungs of ALI mice and enhances the antioxidant capacity by up-regulating the expression of Nrf2 and SOD. It has been reported that excessive production of ROS induced by TWEAK could be eliminated by genetic silencing of Fn14 in macrophages (Madrigal-Matute et al. 2015). Furthermore, TWEAK/Fn14 could promote oxidative stress in the AMPK/PGC1-α-dependent manner (Liu et al. 2018). However, the underlying mechanism remains obscure.

Interestingly, there was no significant increase in TWEAK expression in the lungs of ALI mice (data not shown), indicating that Fn14 may execute its function independent of TWEAK, and there are other ligands for Fn14. Studies have also confirmed that the pro-inflammatory effect of TWEAK is mainly dependent on Fn14. The pro-inflammatory effect of TWEAK disappears after treatment with Fn14-siRNA (Sidler et al. 2017). While, Fn14 may interact with other mediators, mediating tissue inflammation. It has been reported that TWEAK was found to have no significant change in CCl_4_-induced acute liver injury (Wilhelm et al. 2016). While we observed that Fn14 expression was significantly up-regulated in ALI, indicating a pro-inflammatory role in the ALI process.

## Conclusions

Our study indicates that the increased expression of Fn14 is a novel pathological mechanism in ALI. Blocking Fn14 effectively ameliorates LPS-induced ALI in mice. TWEAK/Fn14 axis activates the NLRP3 inflammasome, contributing to the uncontrolled inflammation response in ALI.

## Data Availability

The datasets generated during and/or analyzed during the current study are available from the corresponding author on reasonable request.

## Abbreviations

ALI: Acute lung injury
ARDS: Acute respiratory distress syndrome
ATA: Aurintricarboxylic acid
ATP: Triphosphate
BALF: Bronchoalveolar lavage fluid
BCA: Bicinchoninic acid assay
ELISA: Enzyme-linked immunosorbent assay
Fn14: Fibroblast growth factor-inducible 14
HE: Hematoxylin-eosin
HO-1: Heme oxygenase-1
IL-1β: Interlerkin-1beta
LDH: Lactate dehydrogenase
LPS: Lipopolysaccharide
MCP-1: Monocyte chemoattractant protein-1
MDA: Malondialdehyde
MPO: Myeloperoxidase
NLRP3: NLRs family, pyrin domain containing 3
NF-κB: Nuclear factor-κB
Nrf2: Nuclear factor-erythroid 2-related factor 2
PCR: Real-time polymerase chain reaction
PMVECs: Pulmonary microvascular endothelial cells
ROS: Reactive oxygen species
SOD: Superoxide dismutase
TBA: Thiobarbituric acid
TLR: Toll-like receptors
TNF-α: Tumor necrosis factor-alpha
TWEAK: Tumor necrosis factor-like weak inducer of apoptosis
TBST: Tris-buffered saline
